# Rapid detection of novel coronavirus/Severe Acute Respiratory Syndrome Coronavirus 2 (SARS-CoV-2) by reverse transcription-loop-mediated isothermal amplification

**DOI:** 10.1371/journal.pone.0234682

**Published:** 2020-06-12

**Authors:** Laura E. Lamb, Sarah N. Bartolone, Elijah Ward, Michael B. Chancellor

**Affiliations:** 1 Department of Urology, Beaumont Health System, Royal Oak, Michigan, United States of America; 2 Oakland University William Beaumont School of Medicine, Rochester Hills, Michigan, United States of America; University of Helsinki, FINLAND

## Abstract

Novel Corona virus/Severe Acute Respiratory Syndrome Coronavirus 2 (SARS-CoV-2 or 2019-nCoV), and the subsequent disease caused by the virus (coronavirus disease 2019 or COVID-19), is an emerging global health concern that requires a rapid diagnostic test. Quantitative reverse transcription PCR (qRT-PCR) is currently the standard for SARS-CoV-2 detection; however, Reverse Transcription Loop-Mediated Isothermal Amplification (RT-LAMP) may allow for faster and cheaper field based testing at point-of-risk. The objective of this study was to develop a rapid screening diagnostic test that could be completed in 30–45 minutes. Simulated patient samples were generated by spiking serum, urine, saliva, oropharyngeal swabs, and nasopharyngeal swabs with a portion of the SARS-CoV-2 nucleic sequence. RNA isolated from nasopharyngeal swabs collected from actual COVID-19 patients was also tested. The samples were tested using RT-LAMP as well as by conventional qRT-PCR. Specificity of the RT-LAMP was evaluated by also testing against other related coronaviruses. RT-LAMP specifically detected SARS-CoV-2 in both simulated patient samples and clinical specimens. This test was performed in 30–45 minutes. This approach could be used for monitoring of exposed individuals or potentially aid with screening efforts in the field and potential ports of entry.

## Introduction

The recent Novel Corona virus/Severe Acute Respiratory Syndrome Coronavirus 2 (SARS-CoV-2 or 2019-nCoV) pandemic, and the subsequent disease caused by the virus (coronavirus disease 2019 or COVID-19), has generated global concern given its rapid spread in multiple countries and possible fatal progression of the infection. Initially, many patients reported exposure at a large seafood and animal market in Wuhan, China, suggesting animal-to-person transmission of the virus. However, since then many patients have reported no exposure to animal markets, indicating that person-to-person transmission is occurring. There is currently no vaccine or targeted therapeutic for SARS-CoV-2.

SARS-CoV-2 infection is difficult to diagnose early in infection as patients can remain asymptomatic or present with non-specific clinical symptoms including fever, cough, or shortness of breath. Symptoms may appear in as few as 2 days or up to 2 weeks after exposure [1]. Quantitative reverse transcription PCR (qRT-PCR) for COVID-19 in respiratory samples is currently the standard for diagnostic molecular testing. However, this requires expensive equipment and trained personnel.

Reverse transcription loop-mediated isothermal amplification (RT-LAMP) is a one-step nucleic acid amplification method based on PCR technology that has been used to diagnose infectious diseases [2]. RT-LAMP has several advantages making it attractive for clinical utility. This includes high specificity and sensitivity, and ability to be completed in less than an hour. Furthermore, it can work at various pH and temperature ranges which is advantageous for clinical samples [3]. Lastly, the reagents are relatively inexpensive and can be stable at room temperature. We have previously used RT-LAMP to detect zika virus in clinical serum and urine samples, as well as in crude lysates of single mosquitos [4,5].

This study describes an RT-LAMP methodology that can detect SARS-CoV-2 in simulated patient samples as well as clinical samples in 30–45 minutes. The test could be used at the point-of-care by field and local personnel for the rapid diagnosis of individuals for optimal treatment, isolation, and rapid contact tracing. This methodology could be rapidly developed for the investigation of outbreaks of unknown respiratory diseases where the genetic sequence of the pathogen is known.

## Methods

### RT-LAMP primer design

The consensus sequences of 23 different SARS-CoV-2 strains (EPI_ISL_403929, EPI_ISL_403930, EPI_ISL_403931, EPI_ISL_402129, EPI_ISL_402130, EPI_ISL_402132, EPI_ISL_402128, EPI_ISL_402124, EPI_ISL_402127, EPI_ISL_402121, EPI_ISL_402119, EPI_ISL_402123, EPI_ISL_403962, EPI_ISL_403963, EPI_ISL_404227, EPI_ISL_404228, EPI_ISL_403932, EPI_ISL_403933, EPI_ISL_403934, EPI_ISL_403935, EPI_ISL_403937, EPI_ISL_403936, EPI_ISL_406596, EPI_ISL_406844, EPI_ISL_407079, EPI-ISL_407214) were established when aligned with Lasergene MegAlign (DNASTAR) to identify areas of sequence conservation. To improve specificity, areas of divergence of SARS-CoV-2 (GenBank MN908947) with the sequence-related coronavirus, Bat Severe Acute Respiratory Syndrome (SARS)-like coronavirus (GenBank KY417152.1), were identified using BLAST 2 (Basic Local Alignment Search Tool; NCBI). An area of GenBank MN908947 sequence that had high homology with other SARS-CoV-2 strains and was divergent for Bat SARS-like coronavirus was then targeted for RT-LAMP primer design. The genome equivalent of the SARS-CoV S coding region and Orf8 were purposely avoided. The S coding region was demonstrated to have the most mutations among SARS-CoV isolated strains, suggesting this region may be prone to positive selective pressure, and there were major deletions observed in the Orf8 region of the SARS-CoV genome variants[6]. RT-LAMP primers were designed using LAMP Designer 1.15 (Premier Biosoft), and blasted using Primer-BLAST (NCBI) against genomes of interest. Primer selection was prioritized as described in “A Guide to LAMP primer designing” (https://primerexplorer.jp/e/v4_manual/). In addition, primers were selected to not have four guanines in a row to prevent the formation of tetraplex structures which could disrupt the RT-LAMP reaction. RT-LAMP primers are listed in Table 1 and correspond to the nonstructural protein 3 (NSP3) coding region of open reading frame (ORF) 1Ab. Primer sets include an outer forward primer (F3), outer backward primer (B3), forward inner primer (FIP), backward inner primer (BIP), loop forward primer (LF), and loop backward primer (LB). Primers were ordered from Integrated DNA Technologies and the FIPs and BIPs are HPLC purified.

**Table 1:** RT-LAMP primers for SARS-COV-2 detection.

Primer	Sequence (5’ to 3’)
F3	TCCAGATGAGGATGAAGAAGA
B3	AGTCTGAACAACTGGTGTAAG
FIP(F1c+F2)	AGAGCAGCAGAAGTGGCACAGGTGATTGTGAAGAAGAAGAG
BIP(B1c+B2)	TCAACCTGAAGAAGAGCAAGAACTGATTGTCCTCACTGCC
LoopF	CTCATATTGAGTTGATGGCTCA
LoopB	ACAAACTGTTGGTCAACAAGAC

### Control strain details

SARS-CoV-2 PCR-standard was designed from nucleotide 2941–3420 of the SARS-CoV-2 Wuhan-Hu-1 complete genome (MN908947). The SARS-CoV-2 ssDNA control fragment was synthesized through Invitrogen GeneArt. An oligo of GenBank MN908947.3 was used as there were no commercial sources for a SARS-CoV-2 control plasmid at the time this paper was prepared. The fragment was reconstituted to 10ng/μL and used for LAMP and qPCR experiments. Approximate copy numbers were calculated using a Copy Number calculator [7]. RT-LAMP and PCR controls for Middle East Respiratory Syndrome (MERS), Betacoronavirus England-1 (BtCoV), and Murine hepatitis virus (MHV) were similarity made. We were unable to make an RT-LAMP and a PCR control for any SARS sequences as this is controlled under European Union export regulations.

### Ethics statement

All studies are in compliance with ethical practices and were approved by Beaumont Health’s Institutional Review Board (IRB approval #2020–040 and 2016–044). For studies using samples from healthy participants, the study participants were provided with an information sheet and gave their informed verbal consent to participate, as approved by the IRB. Since samples were deidentified and the study presented no more than minimal risk or harm to the subjects, written consent was not obtained as the principal risk would be potential harm resulting from a breach of confidentiality. The IRB determined that a waiver of consent documentation was appropriate given the study involves no more than minimal risk to human participants per the code of federal regulations. Submission of the sample was interpreted as the participants’ informed consent to participate. For studies using samples from with COVID-19 symptoms, full written consent was obtained and documented in the patients’ medical record. All experiments were performed in accordance with the ethical standards noted in the 1964 Declaration of Helsinki and its later amendments.

### Patient samples

Samples from multiple, consenting, adult volunteers were tested from either fresh or frozen state for each assay. Fresh samples were kept on ice until analysis. For whole mouth saliva and oropharyngeal swabs, participants refrained from eating or drinking anything other than water for 5 minutes prior to collection and rinsed their mouths with water prior to collection. Oropharyngeal swab and nasopharyngeal swabs samples were collected using a Flocked sterile plastic swab applicator which was placed in 3mL of a Universal media for Viruses, Chlamydiae, Mycoplasmas, and Ureaplasmas vial (Bector, Dickinson and Company). To demonstrate our point-of-care RT-LAMP assay and to test possible interference with agents in serum, urine, saliva, oropharyngeal swab, and nasopharyngeal swab collection, various concentrations of SARS-CoV-2 oligo were spiked in samples for test validation. Water was used as a no template control. For direct testing of clinical samples, of RNA (in 100 μL of eluate) was isolated from 210 μL viral transport media from nasopharyngeal swabs using Biomerieux easyMAG and Promega Maxwell (Viral Total Nucleic Acid; AS1330) automated extraction instruments by Beaumont’s Clinical Laboratory Improvement Amendments (CLIA)-licensed Clinical Reference Lab. Patients were confirmed to be SARS-CoV-2 positive or negative by one of the following SARS-CoV-2 qRT-PCR tests: fNxTAG CoV Extended Panel Assay on the Luminex MAGPIX instrument; or the CDC 2019-Novel Coronavirus (2019-nCoV) Real-Time RT-PCR Diagnostic Panel on a Maxwell Instrument. The CDC assay targets two different regions of the N gene (N1 and N2). We tested 30 SARS-CoV-2 positive samples and 30 SARS-CoV-2 negative samples.

### SARS-CoV-2 RT-LAMP primer specificity

Percent mismatch for RT-LAMP primers was determined by aligning the SARS-CoV-2 genome (Wuhan City, Hubei, 2019-12-26, GenBank ID MN908947) to 22 other sequenced SARS-CoV-2 strains from various locations around the world using the Global Initiative on Sharing All Influenza data (GISAID), EpiFlu database and the BLAST Global Alignment tool [8,9]. RT-LAMP primers were also compared to other coronaviruses using the BLAST Global Alignment tool to determine specificity and percent mismatch using the same method. Percent mismatch was calculated using the following equation:

$$\%Mismatch=\frac{Total\#of\;nucleotide\;mismatches\;between\;each\;primer\;and\;sequence}{Total\#of\;nucleotides\;in\;all\;primers}$$

### RT-LAMP

All set-up and execution of RT-LAMP reactions were done in an enclosed room using designated pipettes and filter tips. Analysis and imaging took place in separate rooms to prevent contamination. RT-LAMP reactions were executed in a total volume of 25μL of 1x isothermic amplification buffer, 1.4 mM dNTPs, 8 mM MgSO_4_, 1.6 μM FIP/BIP, 0.2 μM F3/B3, 0.4 μM FL/BL primers, 0.32 U/μL *Bst 2*.*0*, 1 U/μL Antarctic Thermolabile UDG, and 0.6 U/μL *WarmStart Reverse Transcriptase* in ddH_2_0. A total of 2.0 μL of patient sample was used per reaction. Addition of the uracil-DNA glycosylase (UDG) reduced crossover contamination from previous reactions. Reactions were set-up on ice, incubated at 63°C for 30 minutes (15–45 minutes have been tested), and then inactivated at 80°C for 10 minutes. In order to optimize visualization of positive reactions, 2 μL SYBR Green I was added to reactions at a 1:10 dilution. For testing RNA isolated from clinical samples with COVID-19 symptoms, 3 μL of sample were used and reactions were done for 45 minutes. All experiments were replicated 3–5 times.

### qRT-PCR

For testing, a serial dilution of SARS-CoV-2 ssDNA control fragment was made, from 2 ng to 0.06 fg. Nuclease-free ddH_2_O was used as a no template control. No template control or SARS-CoV-2 was added to SYBRSelect Master Mix (ThermoFisher), and 10 μM forward and reverse primers with a total reaction volume of 20 μL. Primers used were F 5’- ATTTGGTGCCACTTCTGCTGC -3’ and R 5’- TCACTGCCGTCTTGTTGACCA -3’. Cycling conditions were 50 °C for 2 minutes and 95 °C for 10 minutes, followed by 40 cycles of 95 °C for 15 seconds and 60 °C for 1 minute, performed on a QuantStudio 3 PCR System (Applied Biosystems).

### Analysis of RT-LAMP

1:10 SYBR green I (Life Technologies) dilution was made in TAE buffer, then 2 μL of the SYBR dilution was added to the RT-LAMP reactions. The visual change of color (orange to yellow) can be used to identify positive amplifications. In addition, the SYBR green I PCR tubes were imaged under UV light in the Bio-Rad ChemiDoc XRS+ Imaging System as the reaction creates a fluorescent output. One half of the RT-LAMP reaction was electrophoresed along with Invitrogen Low DNA Mass Ladder on a 2% agarose gel in 1x TAE buffer (40 mM Tris, 20 mM acetic acid, 1 mM EDTA) at 90 V for 90 minutes. Gels were imaged under UV light using the Bio-Rad ChemiDoc XRS+ Imaging System. Lanes containing a laddered banding pattern were qualified as a positive amplification. Clinical samples were considered positive for RT-LAMP products if they had both a fluorescent signal by excitation of UV light and if they had a laddering pattern when run on a gel.

## Results

### RT-LAMP is specific and sensitive for SARS-CoV-2

**Fig 1:**
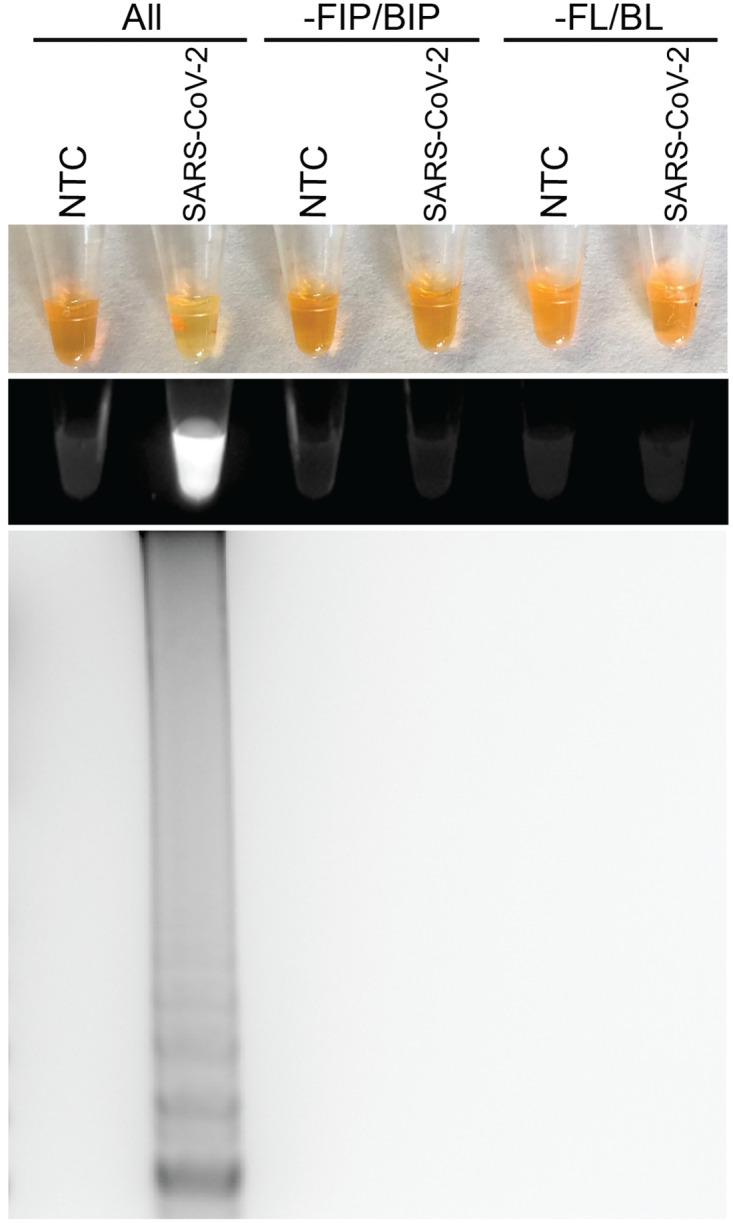
RT-LAMP detection of SARS-CoV-2. (A) SARS-CoV-2 RT-LAMP amplification of SARS-CoV-2 PCR standard (SARS-CoV-2; IDT custom oligo) but not no template control (NTC; negative control) as visualized by addition of SYBR Green I (SYBR) by eye (upper panel), fluorescence (middle panel), or gel electrophoresis (bottom panel). All primers (ALL) are required for effective RT-LAMP reaction. Reactions without FIP and BIP (-FIP/BIP) or FL and BL (-FL/BL) primers resulted in a negative RT-LAMP reaction.

**Fig 2:**
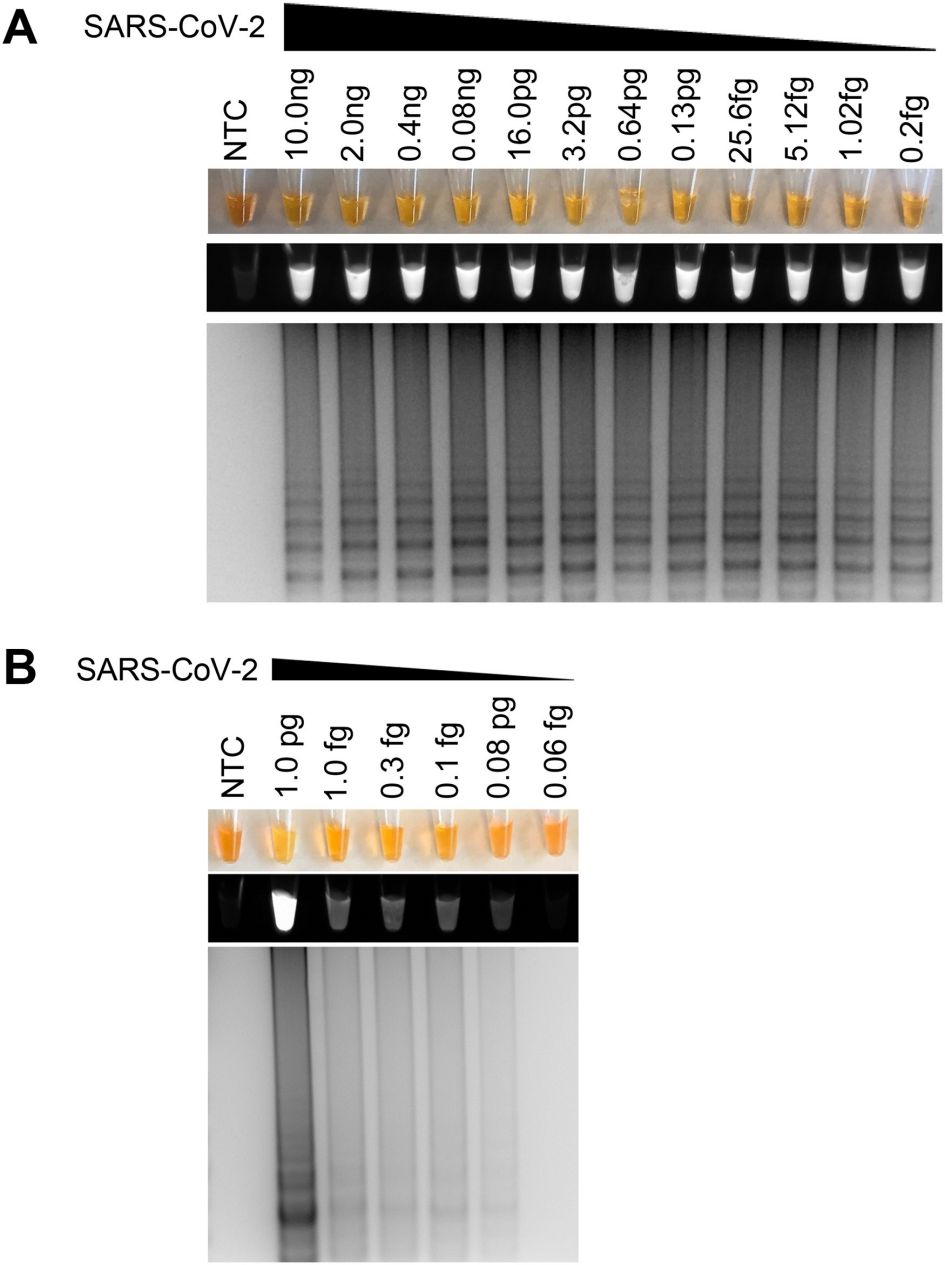
SARS-CoV-2 RT-LAMP sensitivity for SARS-CoV-2. Sensitivity assessment of SARS-CoV-2 RT-LAMP using serial dilutions of SARS-CoV-2 PCR Standard from 10.0 ng/reaction to 0.06 fg/reaction as visualized by addition of SYBR Green I by eye (upper panel), fluorescence (middle panel), or gel electrophoresis (bottom panel). NTC: No template control (negative control).

To establish the optimal conditions for RT-LAMP using a SARS-CoV-2 PCR-standard designed from nucleotide 2941–3420 of the SARS-CoV-2 Wuhan-Hu-1 complete genome (MN908947), several primer sets, ranges of incubation temperatures (57–65°C), and incubation times (15–45 mins) were tested. The best amplification results were obtained at 63°C for 30 minutes as indicated by a banding pattern after electrophoresis on a gel (Fig 1). Positive reactions containing SYBR Green I could be observed by naked eye by a color change from orange to yellow (Fig 1- top panel), with a fluorescent light in response to UV excitation (Fig 1- middle panel), or by a laddering pattern of bands after electrophoresis on a gel (Fig 1-bottom panel). The RT-LAMP reaction required all 6 primers to work under the optimized conditions; removing the forward and backward inner primers or the loop primers did not result in a positive result (Fig 1). In order to determine the lower detection limit of the RT-LAMP reaction for SARS-CoV-2, a dilution series ranging from 10.0 ng to 0.06 fg SARS-CoV-2 DNA olgio was amplified (Fig 2). This corresponds to approximately 3.802×10^10^ to 228 copies of virus. The viral load in patients has been reported to range from 641 copies/mL to 1.34×10^11^ copies/mL, with a median of 7.99×10^4^ in throat samples and 7.52×10^5^ in sputum samples [10]. The limit of detection was estimated to be 0.08 fg, which is approximately equal to 304 viral copies. This dilution series was run in parallel with qRT-PCR using primers that targeted this same region of the SARS-CoV-2 genome; the qRT-PCR Ct values for the dilution series are reported in S1 Table.

### SARS-CoV-2 detection in simulated clinical samples with RT-LAMP

**Fig 3:**
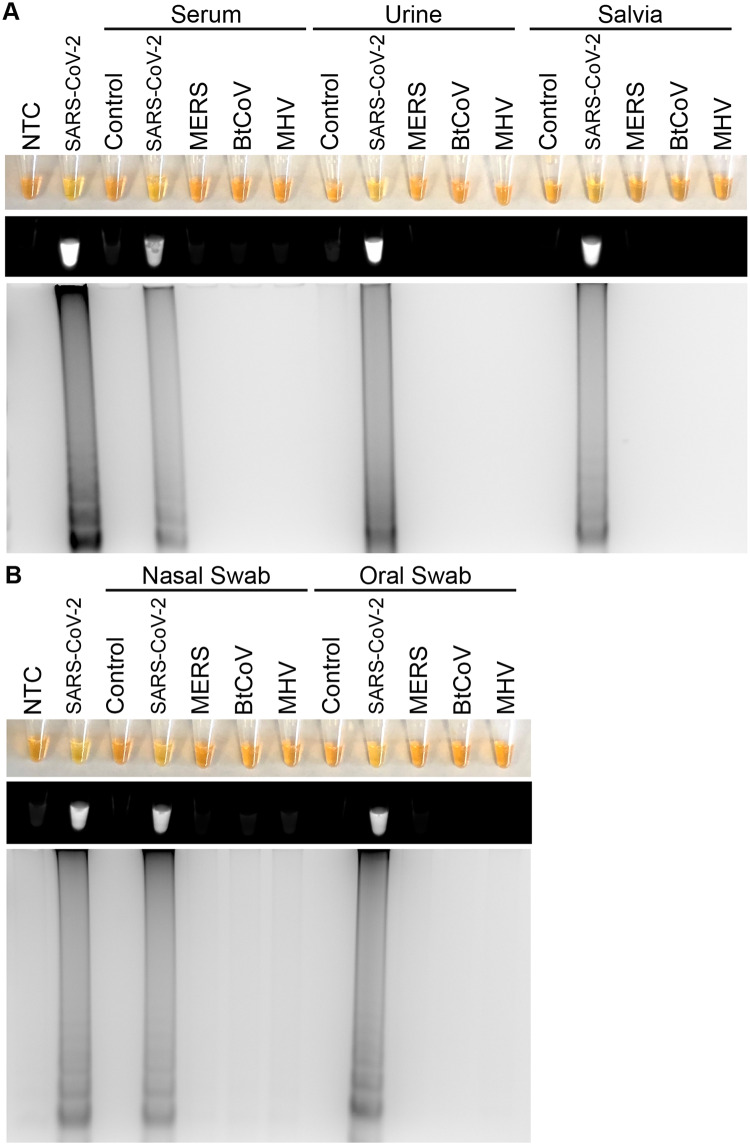
SARS-CoV-2 RT-LAMP specificity for SARS-CoV-2 in simulated patient samples. Specificity assessment of SARS-CoV-2 RT-LAMP in control samples (control) or samples spiked with SARS-CoV-2, MERS, BtCoV, MHV PCR standards (IDT custom oligos) as visualized by the addition of SYBR Green I by eye (upper panel), fluorescence (middle panel), or gel electrophoresis (bottom panel). Types of human samples tested included serum, urine, saliva, nasopharyngeal swabs (nasal swab), or oropharyngeal swabs (oral swab). NTC: No template control (negative control). 3–5 different patient samples were tested for each condition with one representative patient being shown.

To demonstrate the clinical utility of this system for SARS-CoV-2 detection, we spiked various human specimens with SARS-CoV-2, Middle East Respiratory Syndrome (MERS), Betacoronavirus England-1 (BtCoV), or Murine hepatitis virus (MHV) control oligos. MERS, BtCoV and MHV spiked samples were used to test the specificity of the RT-LAMP assay. Spiked specimens included serum, urine, saliva, oropharyngeal swabs, and nasopharyngeal swabs as we wanted to verify that substances present in these samples would not interfere with RT-LAMP. Samples were directly used for RT-LAMP without performing nucleic acid isolation. Only samples containing SARS-CoV-2, but not MERS, BtCoV, or MHV, had positive RT-LAMP reactions indicating specificity of the reaction for SARS-CoV-2 (Fig 3). Furthermore, RT-LAMP could be successfully completed using human serum, urine, saliva, oropharyngeal swabs, and nasopharyngeal swabs.

### RT-LAMP is specific for SARS-CoV-2

The sequences of the RT-LAMP primers were also compared to aligned sequences of various strains of Severe Acute Respiratory Syndrome coronaviruses (SARS) or coronaviruses commonly associated with the common cold (human coronavirus strains 229E, NL63, HKU1, or OC43). These other coronaviruses had 27–54% nucleotide mismatch with our RT-LAMP primers, making it highly unlikely they would give a positive RT-LAMP result, supporting the specificity of this assay for SARS-CoV-2 (Table 2). Given that viruses are prone to genetic mutation, we likewise examined if RT-LAMP primers had any mismatch with 27 different isolated strains of SARS-CoV-2 from various locations. The 27 strains were from different regions of China (Hubei, Zhejiang, and Guangdong providences), France, Thailand, Finland, United States, and Australia. The strains were collected and sequenced from 12/24/2019 to 01/29/2020. Thus these 27 strains reflect more than one region and time point. There was 0% mismatch with all the strains examined, suggesting that these RT-LAMP primers would identify all 27 strains of SARS-CoV-2 examined (S2 Table).

**Table 2:** RT-LAMP primers alignment with other coronaviruses.

Virus	GenBank ID	% Mismatch
SARS-COV-2	MN908947	0
Bat SARS-like CoV 2015	MG772933.1	27·22
Bat SARS-like CoV 2017	MG772934.1	30·77
Bat SARS CoV RM1/2004	KY417144.1	40·83
SARS CoV ZS-C	AY395003.1	42·60
Civet SARS CoV SZ16/2003	AY304488.1	39·64
SARS CoV	NC_004718.3	40·24
SARS CoV MA15	FJ882957.1	40·24
Middle East Respiratory CoV	NC_019843.3	52·07
Betacoronavirus England 1	NC_038294.1	54·44
Murine hepatitis virus	NC_001846.1	53·25
Human Coronavirus 229E	NC_002645.1	51·48
Human Coronavirus NL63	NC_005831.2	54·44
Human Coronavirus HKU1	NC_006577.2	53·25
Human Coronavirus OC43	NC_006213.1	49·11

### RT-LAMP detection of SARS-CoV-2 in clinical specimens

**Fig 4:**
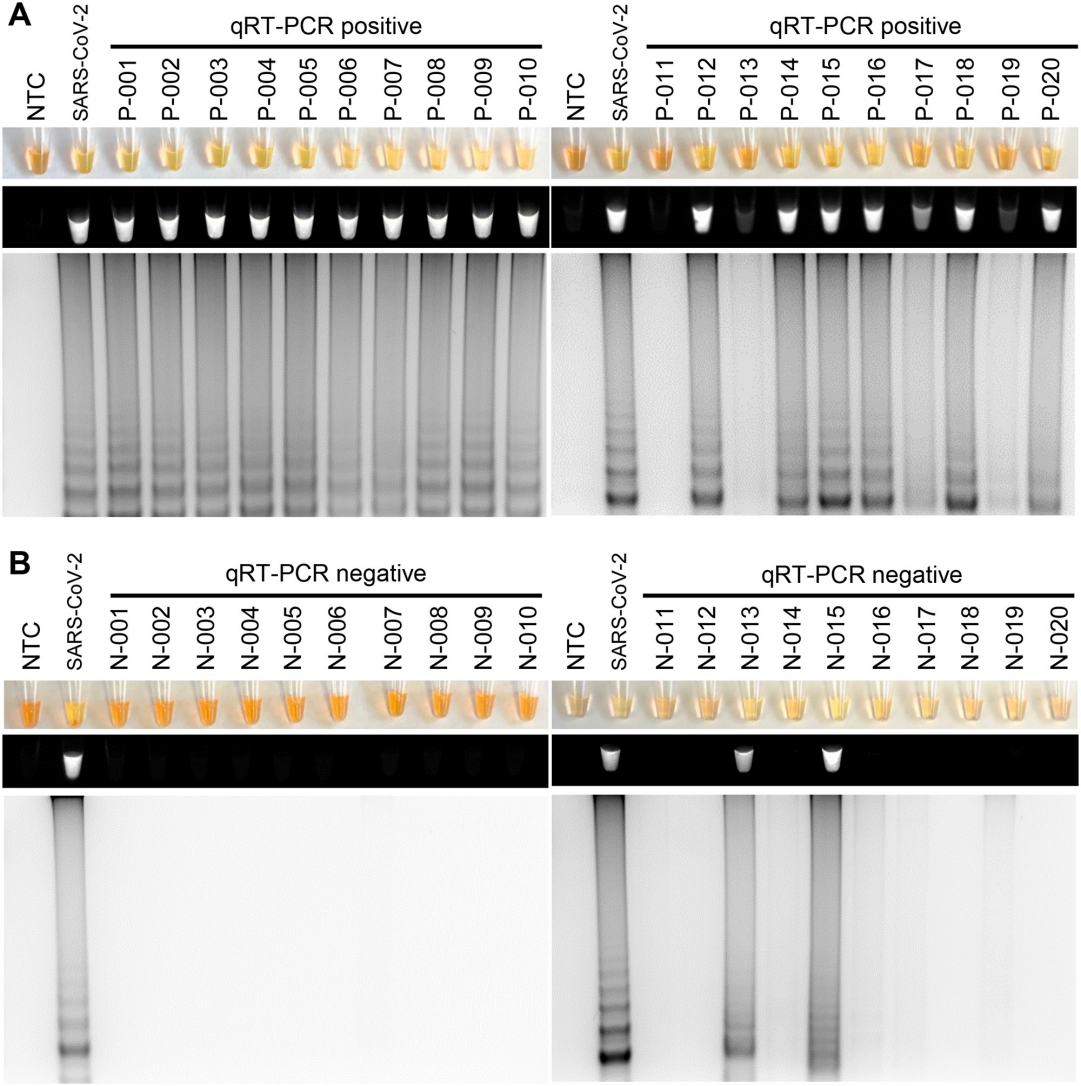
Detection of SARS-CoV-2 with RT-LAMP in patient nasopharyngeal swab samples with RNA isolation. RNA isolated from patients with COVID-19 symptoms with (A) or without (B) SARS-CoV-2 detection by qRT-PCR were tested for SARS-CoV-2 by RT-LAMP. SARS-CoV-2 detection by RT-LAMP was visualized by the addition of SYBR Green I by eye (upper panels), fluorescence (middle panels), or gel electrophoresis (bottom panels).

**Fig 5:**
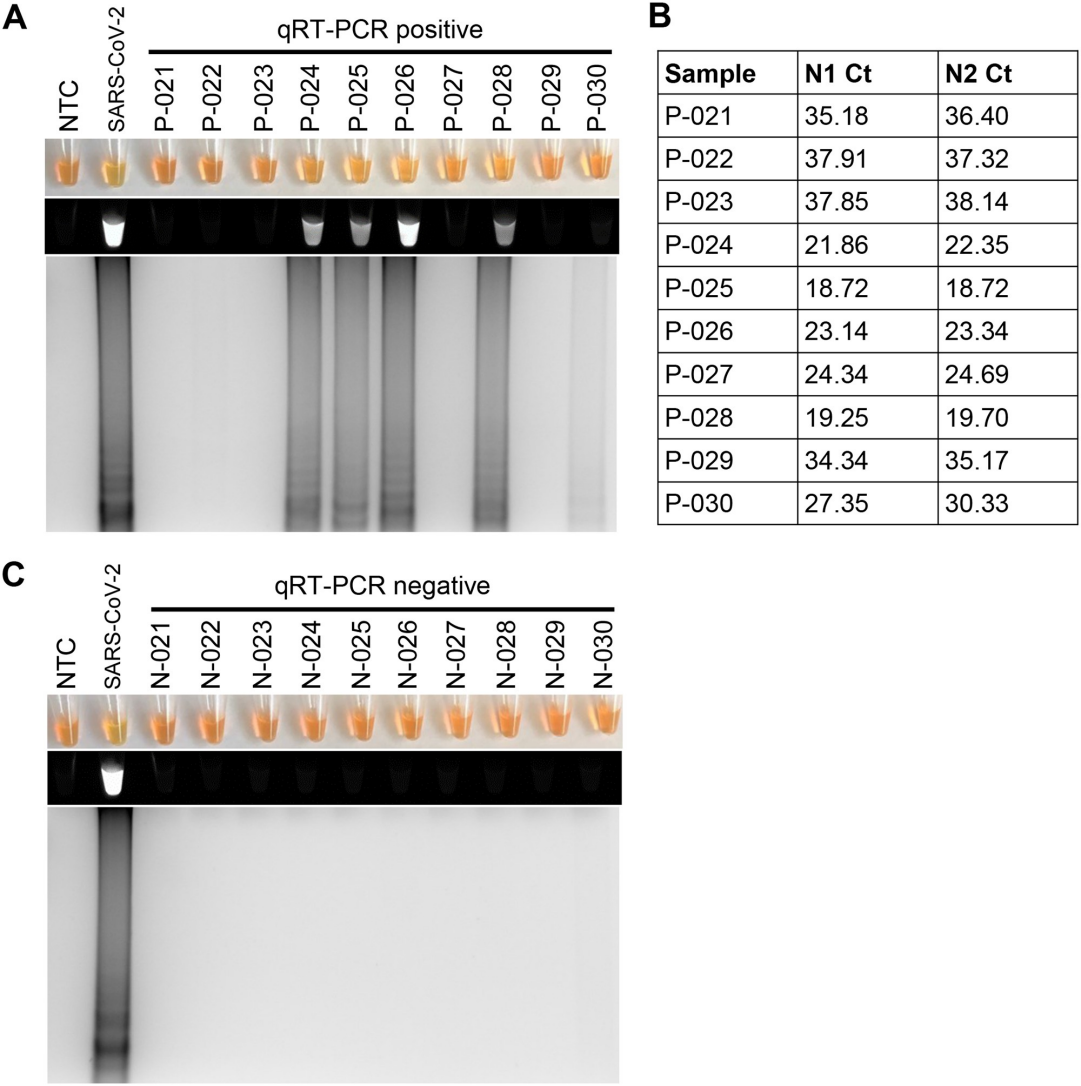
Detection of SARS-CoV-2 with RT-LAMP in patient nasopharyngeal swab samples without RNA isolation. Nasopharyngeal swabs in viral transport media from patients with COVID-19 symptoms with (A) or without (C) SARS-CoV-2 detection by qRT-PCR were tested for SARS-CoV-2 by RT-LAMP. SARS-CoV-2 detection by RT-LAMP was visualized by the addition of SYBR Green I by eye (upper panels), fluorescence (middle panels), or gel electrophoresis (bottom panels). B) Average Ct values from qRT-PCR performed on isolated RNA from the same samples in panel A are listed for target SARS-CoV-2 genes N1 and N2.

We next sought to determine if RT-LAMP could detect SARS-CoV-2 in samples collected from patients with COVID-19. Patients with COVID-19 symptoms were confirmed positive or negative for SARS-CoV-2 using standard clinical qRT-PCR testing. Using the same RNA from the patients that was used for the qRT-PCR testing, RT-LAMP was in agreement with the qRT-PCR results in 95% (N = 19/20) of the SARS-CoV-2 positive by qRT-PCR patients (Fig 4A), and 90% (N = 18/20) of the SARS-CoV-2 negative by qRT-PCR patients (Fig 4B). Given that spiking nasopharyngeal swabs samples in viral transport media with SARS-CoV-2 control oligos was able to work with RT-LAMP (Fig 3), we next tested to determine if RT-LAMP could detect SARS-CoV-2 directly without RNA isolation in these nasopharyngeal swab samples. RT-LAMP detected 40% (N = 4/10) of SARS-CoV-2 positive by qRT-PCR patients, and 100% (N = 10/10) of the SARS-CoV-2 negative by qRT-PCR patients (Fig 5). The samples that were positive by RT-LAMP all had a high level of viremia, as indicated by the Ct values less than 24 Ct by qRT-PCR. In contrast, all the samples negative by RT-LAMP had a Ct value greater than 24 Ct by qRT-PCR.

## Discussion

Given the rapid emergence of SARS-CoV-2 and the severe complications that can result including acute respiratory distress syndrome (ARDS), pneumonia, and even death, improved diagnostic options that are fast, reliable, easy, and affordable are required. This is critical since SARS-CoV-2 infection can rapidly progress from hospital admission to ARDS in as few as 2 days, and SARS-CoV-2 infection can be fatal [1]. qRT-PCR is specific and sensitive. However, qRT-PCR requires trained clinical laboratory personnel to use specialized equipment in a qualified laboratory setting. Since this virus is spreading rapidly and is highly contagious, centralized labs have had trouble keeping up with testing demands in part due to a significant shortage in available qRT-PCR tests despite significant investments by local governments and private industry. This feasibility study demonstrated that RT-LAMP allows rapid detection of SARS-CoV-2 in a variety of common human specimens collected for clinical testing, including serum, urine, saliva, oropharyngeal swabs, and nasopharyngeal swabs.

Currently, clinical testing for SARS-CoV-2 is done by central testing laboratories, which may take one or more days. This study sought to improve upon this by developing a potential point-of-care test. Point-of-care testing has several advantages for SARS-CoV-2. Point-of-care testing should be fast, easy to use by a range of users, low cost, and require little if any laboratory infrastructure. Furthermore, the test needs to be reliable, sensitive, and specific. RT-LAMP meets these requirements and therefore has large value for screening and testing for SARS-CoV-2 in potentially exposed populations. Other groups are working to adopt RT-LAMP for SAR-CoV-2 into alternative readouts. For example, the primers designed by our group along with two other primer sets were adopted for fluorogenic oligonucleotide strand exchange (OSD) probes [11]. This method could allow multiplexing and thus more than one genomic target of SARS-CoV-2 to be included in an assay. Furthermore, this group also demonstrated that the RT-LAMP primers described here and modified for OSD are compatible for RT-LAMP with the Bst DNA polymerase large fragment (Bst-LF), which possesses reverse transcriptase ability as well as DNA polymerase activity. This detected down to 100 SARS-CoV-2 genomes [11] and Bst-LF can be made by bacteria without a purification step, useful for situations with an interrupted supply chain or in settings that are resource poor. RT-LAMP, including the primers described here, are also currently being adopted for LAMP-Seq [12]. This could allow large scale processing using barcoding and next-generation sequencing to pool samples and subsequently identify positive individuals.

RT-LAMP was sensitive for detection of SARS-CoV-2. We were able to detect down to the equivalent of approximately 304 viral copies. Bharda et al have also reported that use of all six of our RT-LAMP primers can detect down to 100 copies of SARS-CoV-2 genomic RNA [11]. The viral load in patients has been reported to range from 641 copies/mL to 1.34×10^11^ copies/mL, with a median of 7.99×10^4^ in throat samples and 7.52×10^5^ in sputum samples [10]. As such, RT-LAMP is sensitive enough for detection of SARS-CoV-2 in clinical samples.

We sought to determine if this RT-LAMP assay worked in a range of different samples that might be collected in a clinical setting or as a possible non-invasive screening tool. This was important to determine since it may not be feasible to always collect certain specimens. For example, swabs used for nasopharyngeal sampling as well as viral transport media has experienced shortages recently. Although SARS-CoV-2 has been reported to not be detectable in serum of all patients [13], serum may also be difficult to collect from patients who are critically sick or dehydrated, or from elderly, children, and neonates. Furthermore, not all biological samples may be compatible with RT-LAMP. Some sample types may contain chemicals that inhibit nucleic acid assays if the sample is tested directly without first isolating the RNA. RT-LAMP worked in all the human sample types tested when SARS-CoV-2 control olgios were spiked into the samples. We tested this in samples collected from several individuals to control for variation in sample composition across different people. We also demonstrated that RT-LAMP has similar performance to qRT-LAMP in detecting SARS-CoV-2 in RNA isolated from clinical nasopharyngeal samples. Overall, RT-LAMP was positive for 95% (N = 19/20) of samples positive by qRT-PCR and RT-LAMP was also negative for 90% (N = 18/20) of samples negative by qRT-PCR. The two samples that were positive by RT-LAMP but negative by qRT-PCR could represent false positives, contamination, or increased sensitivity of RT-LAMP compared to qRT-PCR. While RT-LAMP can be prone to false positives, we have taken several precautions to control for this in our assay set-up and false positives will usually not be positive if the samples are tested again. On retesting these samples, they were still positive which suggests that they are not false positives. While we use good laboratory practice to reduce contamination, we cannot exclude this possibility. Lastly, it is possible that RT-LAMP can be more sensitive than qRT-PCR as this has been previously described, although one of the samples that was positive by qRT-PCR was negative by RT-LAMP [14].

We previously demonstrated that RT-LAMP for ZIKV does not require prior RNA isolation from urine or serum samples [4,5]. The use of unprocessed samples could save considerable time and reduce costs. Therefore, we tested RT-LAMP with nasopharyngeal swabs placed in viral transport media directly without an RNA isolation step. In order to detect low levels of viremia, we increased the sample volume added to the RT-LAMP reaction from 2–3 μL and also increased the RT-LAMP incubation time from 30 to 45 minutes. RNA was also isolated from these samples to verify which samples contained SARS-CoV-2 by qRT-PCR. RT-LAMP detected 40% (N = 4/10) of SARS-CoV-2 positive by qRT-PCR patients, and was negative for 100% (N = 10/10) of the SARS-CoV-2 samples also negative by qRT-PCR. The samples that were positive by RT-LAMP all had a high level of viremia, as indicated by the Ct values less than 24 Ct by qRT-PCR. This data suggests that the viral transport media may contain inhibitor(s), possibly glucose, that make RT-LAMP less effective. Hence, RT-LAMP was only able to detect SARS-CoV-2 in samples with high levels of virus. For clinical utility, RT-LAMP has better performance in RNA purified samples compared to direct testing of nasopharyngeal swabs placed in viral transport media. It may be that direct testing of nasopharyngeal swabs is possible for clinical utility by RT-LAMP if the nasal swabs are placed in an alternative matrix, such as saline, water, or ideally a preservative that can stabilize the RNA and render the virus inactive. The potential for direct RT-LAMP testing of other sample types, such as saliva, is also possible.

Primers were designed for a conserved span of SARS-CoV-2 sequence that was found in 27 isolated SARS-CoV-2 strains but was a sequence that was also divergent from related coronavirus SARS. Given that all the isolated strains of SARS-CoV-2 thus far have shown very little genetic differences, we anticipate that this RT-LAMP will detect SARS-CoV-2 with the same level of confidence as qRT-PCR. As with any molecular test, including PCR, it is possible that viral evolution may impact primer binding, but this can be monitored as new strains are sequenced. Of the mutations thus far reported as of April 5, 2020 [15], none of the reported mutations corresponded to the sequence targeted by these RT-LAMP primers. This SARS-CoV-2 RT-LAMP assay was highly specific as it did not give a positive result for MERS, BtCoV, and MHV. The RT-LAMP primers also had significant mismatch with numerous strains of SARS (27–40% mismatch) and common cold associated human coronavirus strains 229E, NL63, HKU1, or OC43 (49–54% mismatch), making it highly unlikely to have cross-reactivity with these other respiratory viruses. We tested a range of temperatures from 57°C to 65°C; all the temperatures gave comparable results, indicating that RT-LAMP for SARS-CoV-2 has a large working temperature range with an optimal temperate of 63°C. We also tested a range of incubation times from 15–45 minutes. The optimal time for detection of RT-LAMP products was 30–45 minutes, however SARS-CoV-2 RT-LAMP products were detectable by UV light excitation or banding patterns on gels in as little as 15 minutes.

This study has several limitations. First, SARS-CoV-2 is Biosafety level 3 so our laboratory was unable to work directly with purified virus. As such, most of the experiments presented here used a nucleotide oligo of SARS-CoV-2 corresponding to the GenBank MN908947 sequence. Similarly, we were unable to directly test related coronaviruses and instead used nucleotide oligos from the same region of those viruses. The primers were further evaluated for specificity by BLASTing them to related coronavirus sequences. However, we have demonstrated that RT-LAMP can detect SARS-CoV-2 in RNA isolated from nasopharyngeal swab samples at a similar detection rate with qRT-PCR. Furthermore, RT-LAMP could detect SARS-CoV-2 in some nasopharyngeal swab samples directly without an RNA isolation step. This does represent a proof-of-feasibility for this assay, in that these RT-LAMP primers can detect SARS-CoV-2 in clinical samples and should be highly specific for SARS-CoV-2 and not detect other related viruses. Although RT-LAMP reactions are highly specific, it is not a quantitative test. Other groups are working at improving the read outs of RT-LAMP assays including the use of smartphone-integrated sensors to make interpretation of the assay even more user-friendly [16]. RT-LAMP reactions can have a higher rate of false positives compared to qRT-PCR. As such, we took several precautions including having a lateral work flow for experiments, using filter pipet tips, and including a Thermolabile Uracil-DNA Glycosylase (UDG) in all reactions to prevent possible carry-over contamination of RT-LAMP products from previous reactions. This study was not powered to determine sensitivity in a clinical population. Lastly, this is a rapidly developing area of study and all the information presented at the time of publication represent the authors’ best knowledge at the time. As our understanding of SARS-CoV-2 continues to develop, this information may change.

## Conclusions

Here we describe a fast and robust assay for detection of SARS-CoV-2 in 30–45 minutes. This simple assay could be used outside of a central laboratory on various types of biological samples. This assay can be completed by individuals without specialty training or equipment and may provide a new diagnostic strategy for combatting the spread of SARS-CoV-2 at the point-of-risk.

## Supporting information

S1 Fig Original images for all gels reported in figures. NTC = no template control; MWM = molecular weight marker; X = lane not included in final image.
(PDF)

S1 Table Detection of SARS-COV-2 at different concentrations by qRT-PCR. Data shown as mean ± standard deviation.
(DOCX)

S2 Table RT-LAMP primers alignment with different isolated strains of SARS-COV-2. (DOCX)
