# Supplementary material for: Rapid detection of novel coronavirus/Severe Acute Respiratory Syndrome Coronavirus 2 (SARS-CoV-2) by reverse transcription-loop-mediated isothermal amplification
Source: PLoS One. 2020 Jun 12;15(6):e0234682. doi: 10.1371/journal.pone.0234682 (PMC7292379; doi:10.1371/journal.pone.0234682)

Supplementary Information - Original images for blots and gels

Rapid Detection of Novel Coronavirus/Severe Acute Respiratory Syndrome Coronavirus 2 (SARS-CoV-2) by Reverse Transcription-Loop-Mediated Isothermal Amplification

Laura E. Lamb[1,2*], Sarah N. Bartolone[1], Elijah Ward[1], Michael B. Chancellor[1,2]

[1] Department of Urology, Beaumont Health System, Royal Oak, MI, United States of America

[2] Oakland University William Beaumont School of Medicine, Rochester Hills, MI, United States of America

*Corresponding author: Laura E. Lamb, PhD
laura.lamb@beaumont.org

ORIGINAL IMAGE FOR FIGURE 1

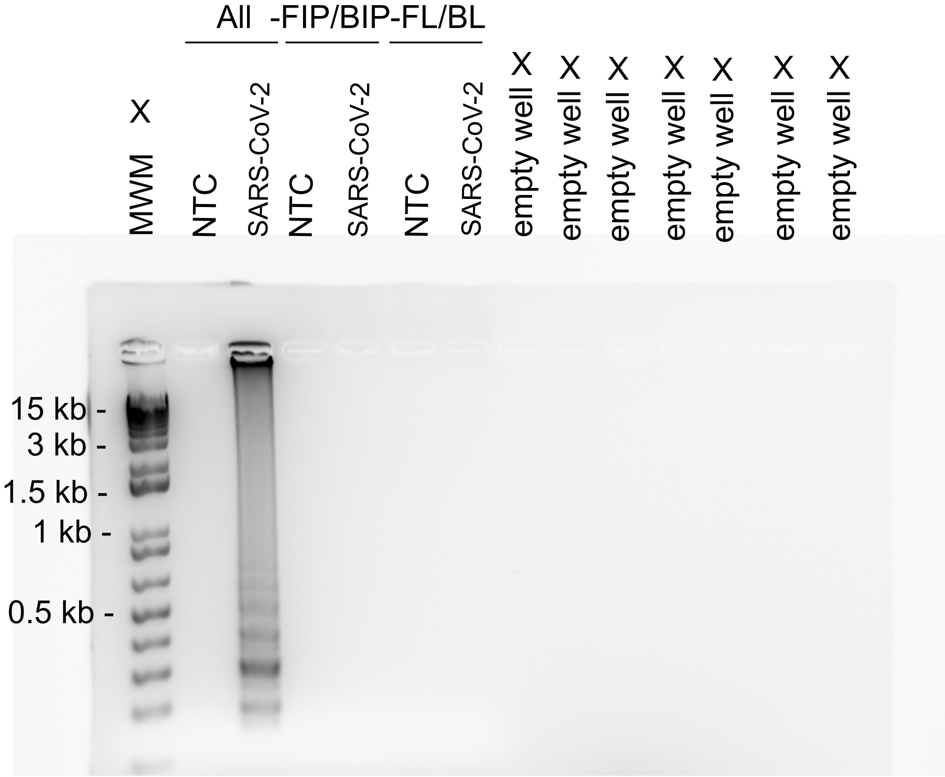

ORIGIAL IMAGES FOR FIGURE 2

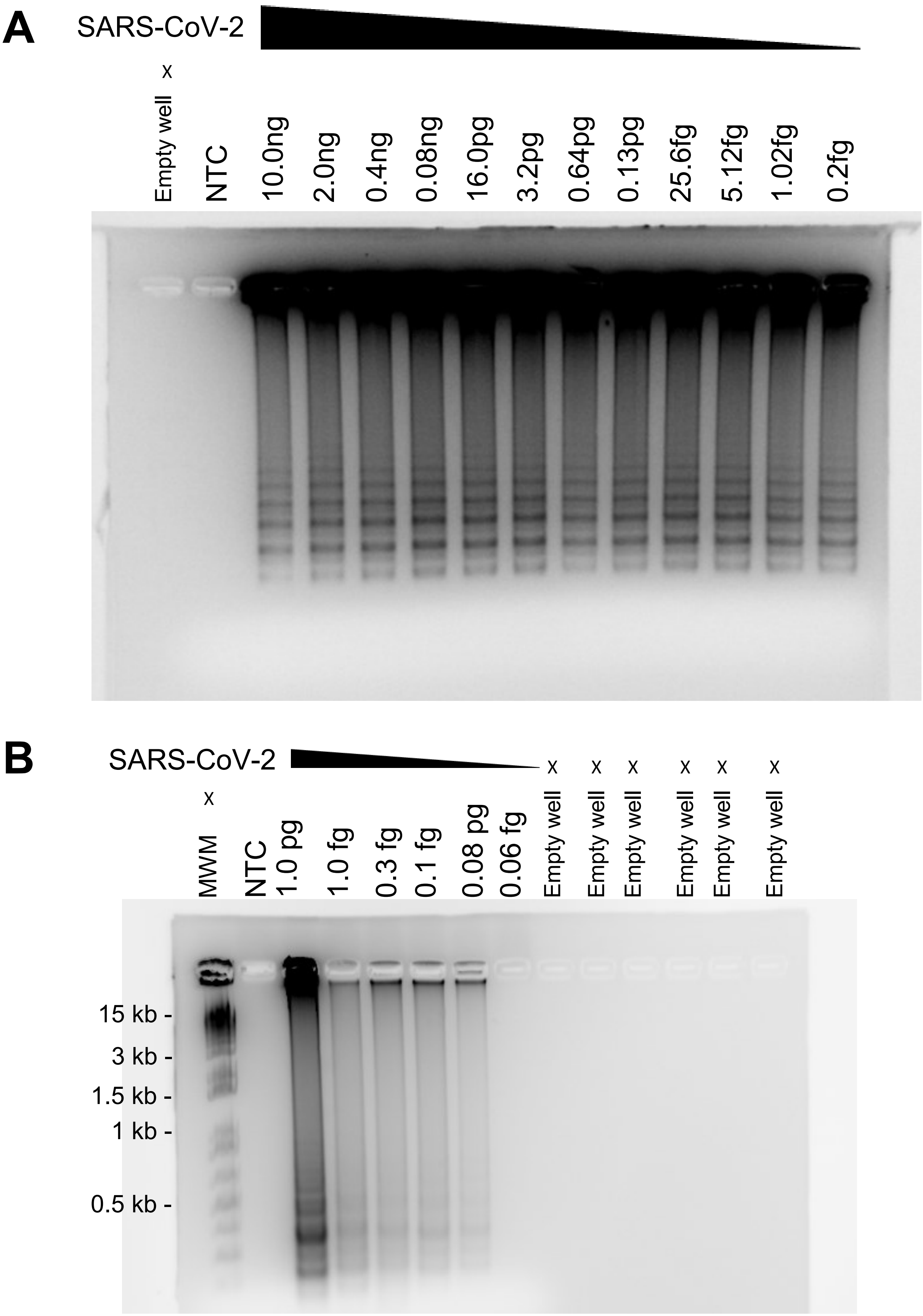

ORIGINAL IMAGES FOR FIGURE 3

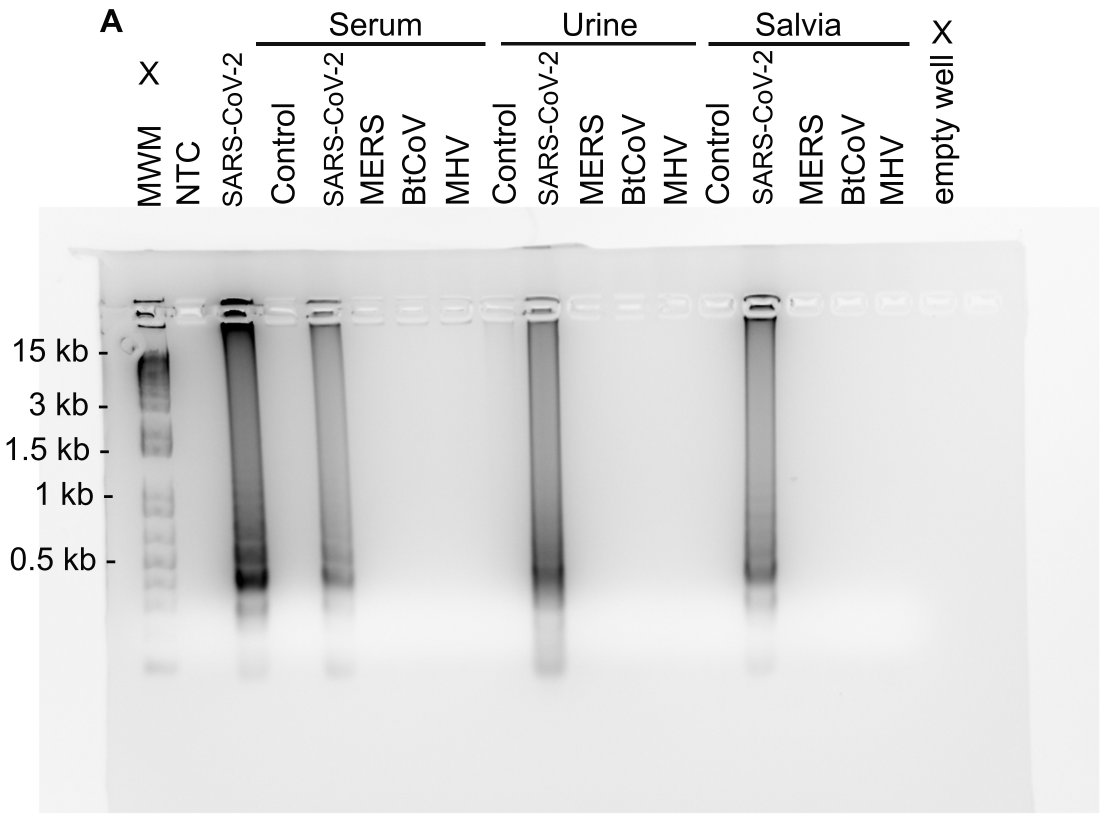

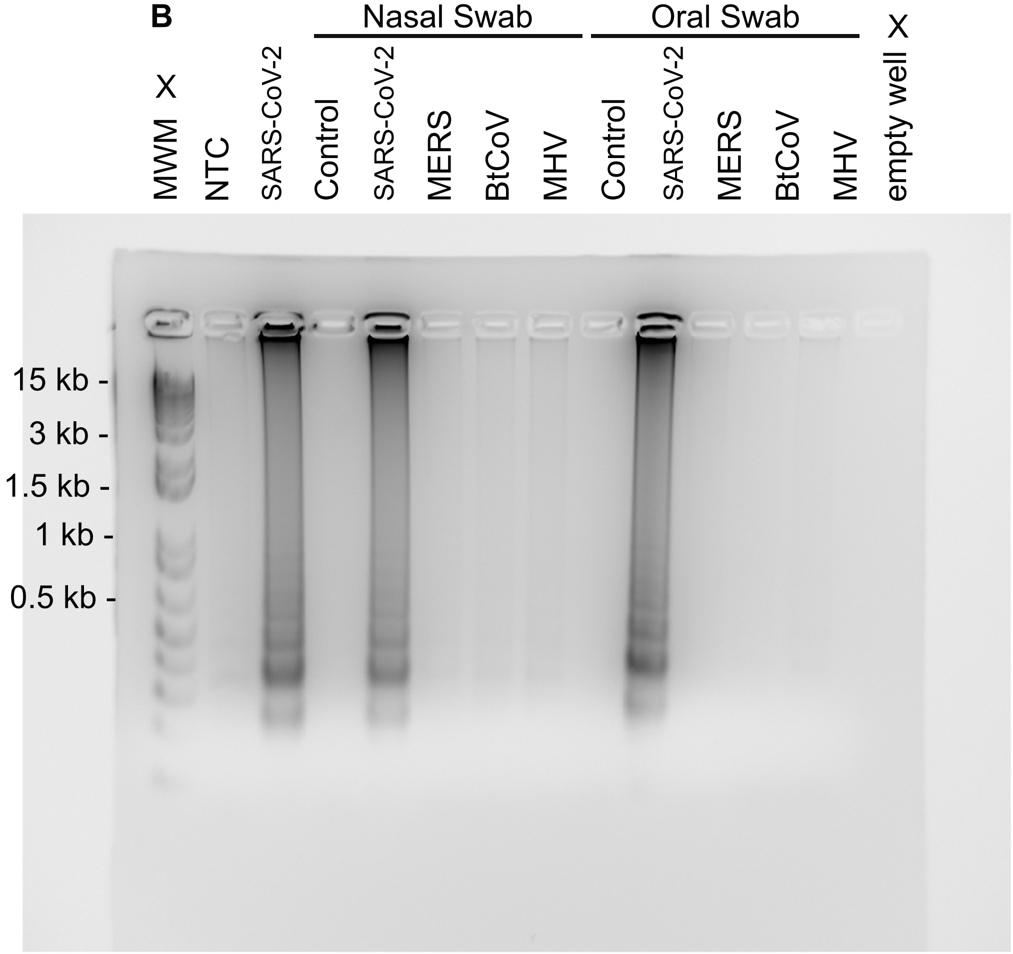

ORIGINAL IMAGES FOR FIGURE 4

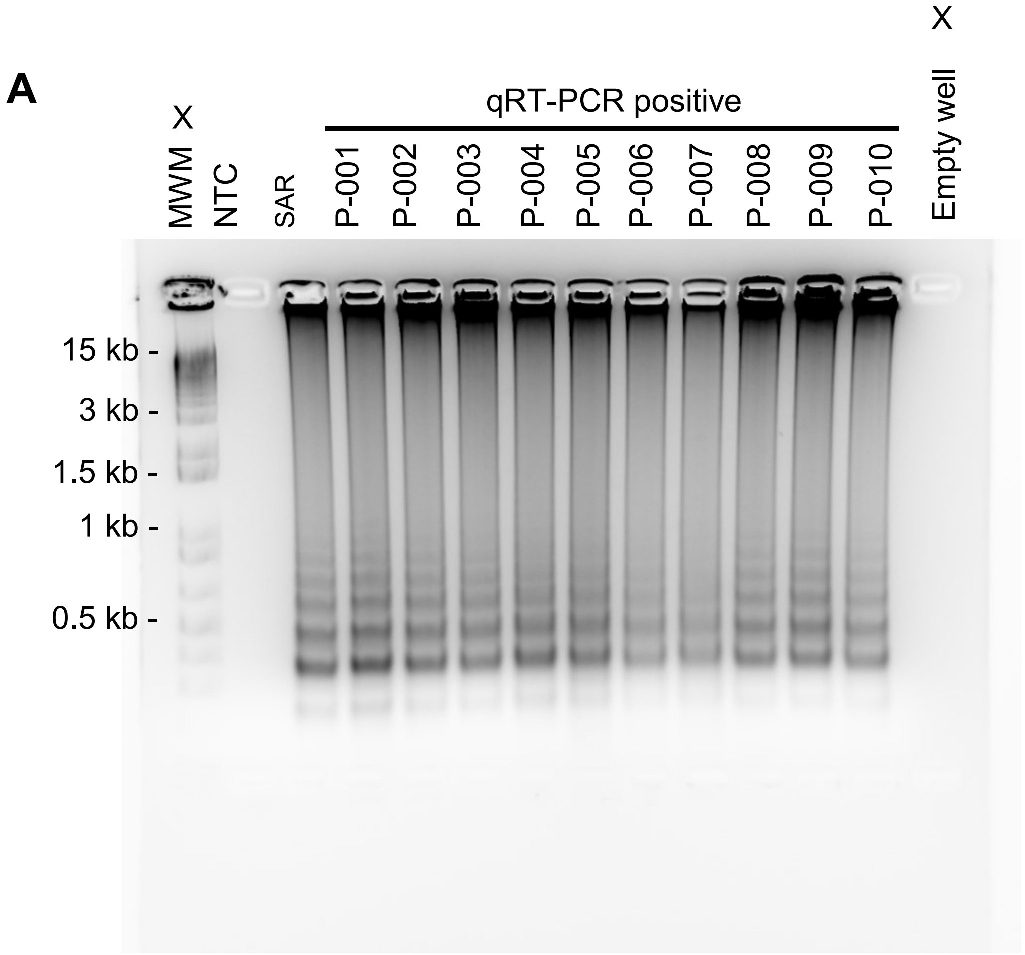

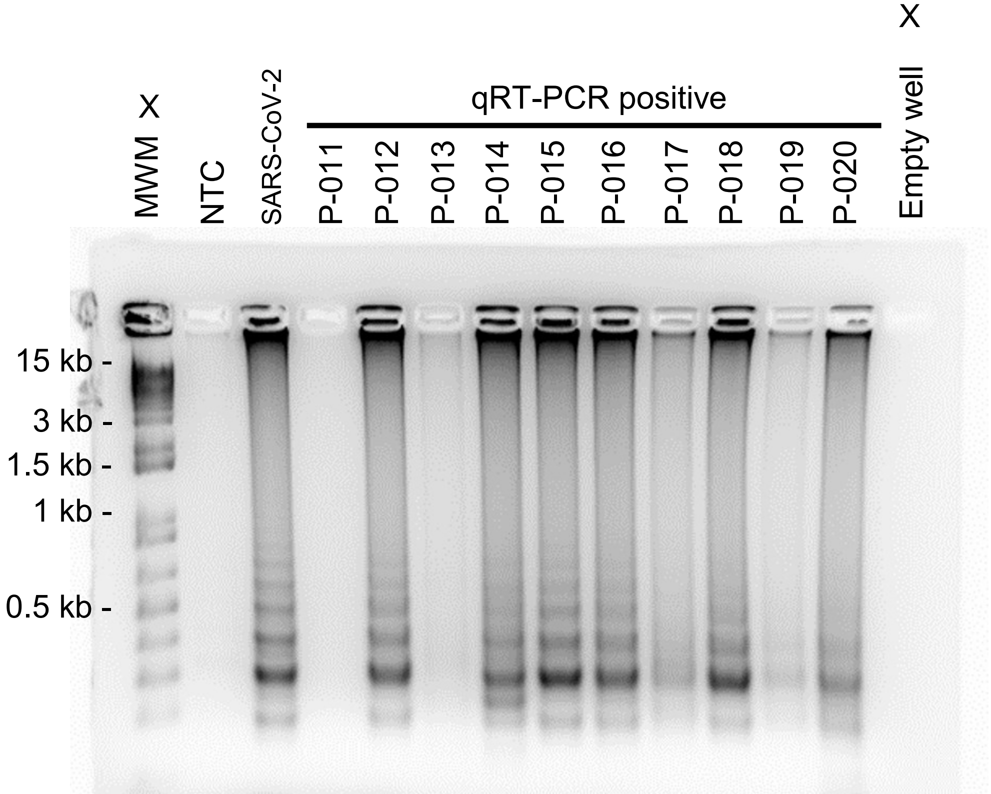

ORIGINAL IMAGES FOR FIGURE 4

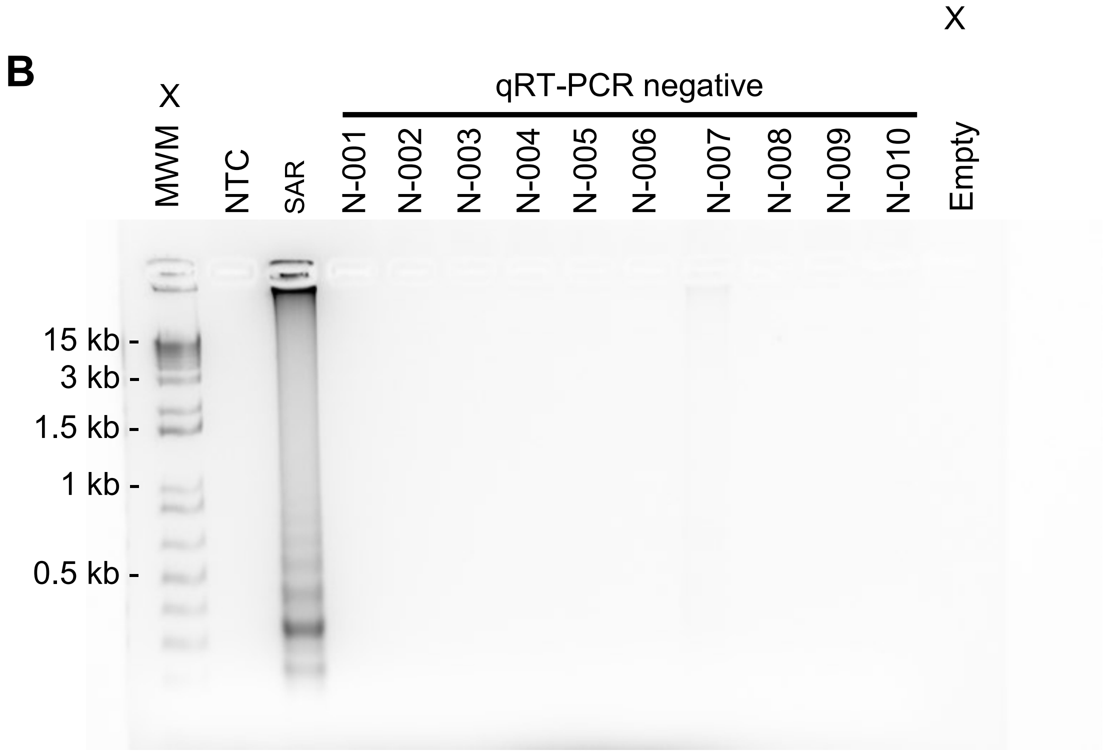

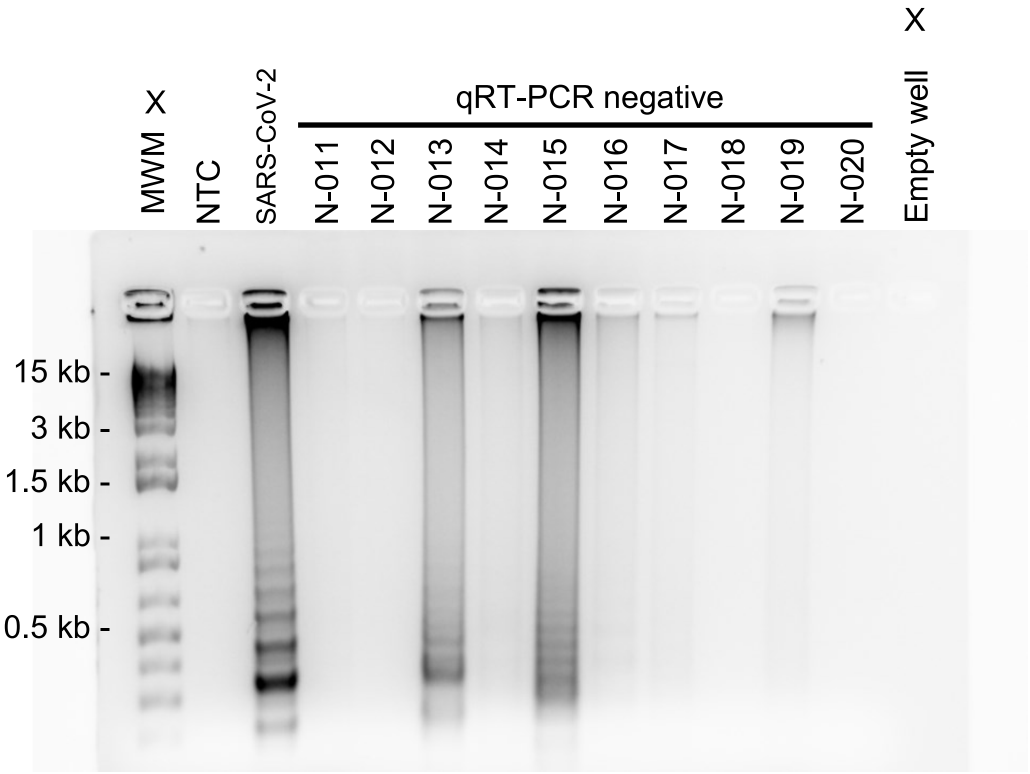

ORIGINAL IMAGES FOR FIGURE 5

A

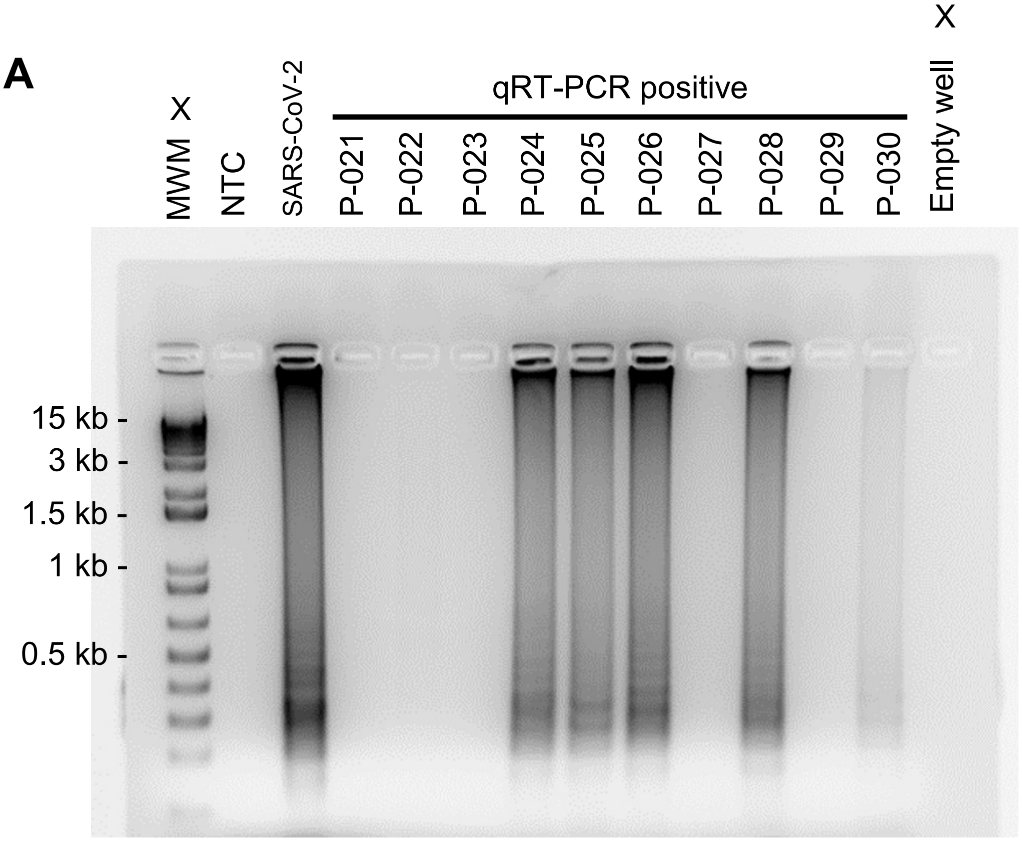

C

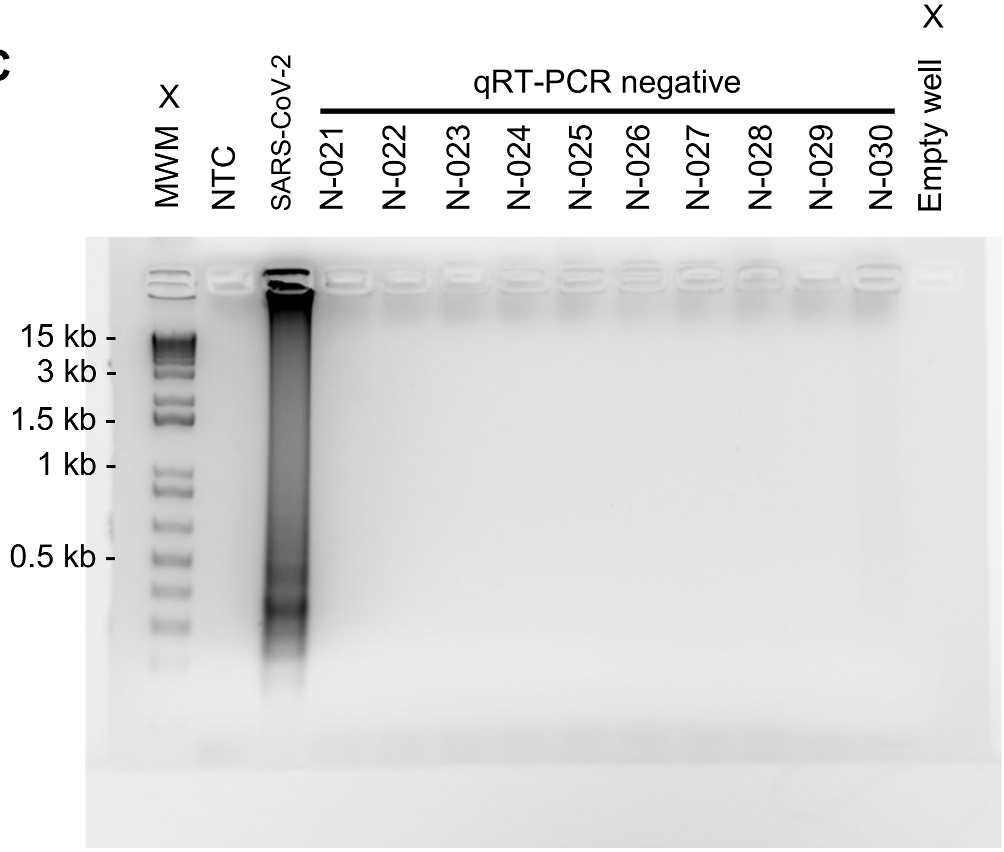

Supplement: S1 Fig — NTC = no template control; MWM = molecular weight marker; X = lane not included in final image. (PDF) [file pone.0234682.s001.pdf]
