# Supplementary material for: Rapid detection of novel coronavirus/Severe Acute Respiratory Syndrome Coronavirus 2 (SARS-CoV-2) by reverse transcription-loop-mediated isothermal amplification
Source: PLoS One. 2020 Jun 12;15(6):e0234682. doi: 10.1371/journal.pone.0234682 (PMC7292379; doi:10.1371/journal.pone.0234682)
Supplement: S1 Table — Data shown as mean ± standard deviation. (DOCX) [file pone.0234682.s002.docx]

**S1 Table. Detection of SARS-COV-2 at different concentrations by qRT-PCR.**

| **Concentration** | **Ct Mean** |
| --- | --- |
| Fig 2a | |
| 2.000 ng | 4.9 |
| 0.400 ng | 6.7 |
| 0.080 ng | 9.1 |
| 0.016 ng | 11.8 |
| 3.200 pg | 14.2 |
| 0.640 pg | 16.7 |
| 0.128 pg | 19.4 |
| 25.600 fg | 21.7 |
| 5.120 fg | 24.1 |
| 1.020 fg | 26.5 |
| 0.204 fg | 29.0 |
| Fig 2b | |
| 1.00 pg | 16.6 |
| 1.00 fg | 27.0 |
| 0.30 fg | 28.3 |
| 0.10 fg | 30.2 |
| 0.08 fg | 30.3 |
| 0.06 fg | 30.7 |

Data shown as mean ± standard deviation.
