# Supplementary material for: Rapid detection of novel coronavirus/Severe Acute Respiratory Syndrome Coronavirus 2 (SARS-CoV-2) by reverse transcription-loop-mediated isothermal amplification
Source: PLoS One. 2020 Jun 12;15(6):e0234682. doi: 10.1371/journal.pone.0234682 (PMC7292379; doi:10.1371/journal.pone.0234682)
Supplement: S2 Table — (DOCX) [file pone.0234682.s003.docx]

**S2 Table. RT-LAMP primers alignment with different isolated strains of SARS-COV-2**

| **Strain & Collection Date** | **GISAID ID** | **% Mismatch** |
| --- | --- | --- |
| Wuhan, Hubei, 2019-12-26 | GenBank MN908947 | 0 |
| Wuhan, Hubei, 2019-12-30 | EPI_ISL_403929 | 0 |
| Wuhan, Hubei, 2019-12-30 | EPI_ISL_403930 | 0 |
| Wuhan, Hubei, 2019-12-30 | EPI_ISL_403931 | 0 |
| Wuhan, Hubei, 2019-12-30 | EPI_ISL_402129 | 0 |
| Wuhan, Hubei, 2019-12-30 | EPI_ISL_402130 | 0 |
| Wuhan, Hubei, 2019-12-30 | EPI_ISL_402132 | 0 |
| Wuhan, Hubei, 2019-12-30 | EPI_ISL_402128 | 0 |
| Wuhan, Hubei, 2019-12-30 | EPI_ISL_402124 | 0 |
| Wuhan, Hubei, 2019-12-30 | EPI_ISL_402127 | 0 |
| Wuhan, Hubei, 2019-12-30 | EPI_ISL_402121 | 0 |
| Wuhan, Hubei, 2019-12-30 | EPI_ISL_402119 | 0 |
| Wuhan, Hubei, 2019-12-24 | EPI_ISL_402123 | 0 |
| Nonthaburi, Thailand, 2020-01-06 | EPI_ISL_403962 | 0 |
| Nonthaburi, Thailand, 2020-01-13 | EPI_ISL_403963 | 0 |
| Zheijang province, 2020-01-16 | EPI_ISL_404227 | 0 |
| Zheijang province, 2020-01-17 | EPI_ISL_404228 | 0 |
| Shenzen, Guangdong, 2020-01-14 | EPI_ISL_403932 | 0 |
| Shenzen, Guangdong, 2020-01-15 | EPI_ISL_403933 | 0 |
| Shenzen, Guangdong, 2020-01-15 | EPI_ISL_403934 | 0 |
| Shenzen, Guangdong, 2020-01-15 | EPI_ISL_403935 | 0 |
| Zhuhai, Guangong, 2020-01-16 | EPI_ISL_403937 | 0 |
| Zhuhai, Guangong, 2020-01-17 | EPI_ISL_403936 | 0 |
| Paris, France, 2020-01-23 | EPI_ISL_406596 | 0 |
| Victoria, Australia, 2020-01-25 | EPI_ISL_406844 | 0 |
| Lapland, Finland, 2020-01-29 | EPI_ISL_407079 | 0 |
| Washington, USA, 2020-01-25 | EPI-ISL_407214 | 0 |
